# Supplementary material for: Resolving the full spectrum of human genome variation using Linked-Reads
Source: Genome Res. 2019 Apr;29(4):635–45. doi: 10.1101/gr.234443.118 (PMC6442396; doi:10.1101/gr.234443.118)
Supplement: Supplemental Material [file supp_gr.234443.118_Supplemental_longranger-2.2.zip › longranger-2.2.tar.bz2!/lr2.2-public/lib/stan/cmdstan/stan/doc/api/html/functions_func_r.html]

Stan: Class Members - Functions


|  |  |
| --- | --- |
|  | Stan  2.5.0  probability, sampling & optimization |


- Main Page
- Related Pages
- Namespaces
- Classes
- Files

- Class List
- Class Index
- Class Hierarchy
- Class Members

- All
- Functions
- Variables
- Typedefs
- Enumerator
- Related Functions

- a
- b
- c
- d
- e
- f
- g
- h
- i
- j
- k
- l
- m
- n
- o
- p
- q
- r
- s
- t
- u
- v
- w
- x
- z
- ~


All Classes Namespaces Files Functions Variables Typedefs Enumerations Enumerator Friends Macros Pages

### - r -

- range()
  : stan::gm::range
- read\_adaptation()
  : stan::io::stan\_csv\_reader
- read\_header()
  : stan::io::stan\_csv\_reader
- read\_metadata()
  : stan::io::stan\_csv\_reader
- read\_samples()
  : stan::io::stan\_csv\_reader
- reader()
  : stan::io::reader< T >
- recorded()
  : stan::common::recorder::sum\_values
- recover\_all()
  : stan::memory::stack\_alloc
- recover\_nested()
  : stan::memory::stack\_alloc
- reject\_statement()
  : stan::gm::reject\_statement
- rel\_grad\_norm()
  : stan::optimization::BFGSMinimizer< FunctorType, QNUpdateType, Scalar, DimAtCompile >
- rel\_obj\_decrease()
  : stan::optimization::BFGSMinimizer< FunctorType, QNUpdateType, Scalar, DimAtCompile >
- remove()
  : stan::gm::variable\_map
  , stan::io::dump
  , stan::json::json\_data
- reserve()
  : validate\_identifier
- reset\_sigs()
  : stan::gm::function\_signatures
- restart()
  : stan::mcmc::base\_adaptation
  , stan::mcmc::stepsize\_adaptation
  , stan::mcmc::windowed\_adaptation
  , stan::prob::welford\_covar\_estimator
  , stan::prob::welford\_var\_estimator
- return\_statement()
  : stan::gm::return\_statement
- returns\_type\_vis()
  : stan::gm::returns\_type\_vis
- round\_error()
  : std::numeric\_limits< stan::agrad::fvar< T > >
  , std::numeric\_limits< stan::agrad::var >
- row\_vector()
  : stan::io::reader< T >
- row\_vector\_constrain()
  : stan::io::reader< T >
- row\_vector\_lb()
  : stan::io::reader< T >
- row\_vector\_lb\_constrain()
  : stan::io::reader< T >
- row\_vector\_lb\_unconstrain()
  : stan::io::writer< T >
- row\_vector\_lub()
  : stan::io::reader< T >
- row\_vector\_lub\_constrain()
  : stan::io::reader< T >
- row\_vector\_lub\_unconstrain()
  : stan::io::writer< T >
- row\_vector\_ub()
  : stan::io::reader< T >
- row\_vector\_ub\_constrain()
  : stan::io::reader< T >
- row\_vector\_ub\_unconstrain()
  : stan::io::writer< T >
- row\_vector\_unconstrain()
  : stan::io::writer< T >
- row\_vector\_var\_decl()
  : stan::gm::row\_vector\_var\_decl
- rows()
  : stan::math::LDLT\_factor< stan::agrad::var, R, C >
  , stan::math::LDLT\_factor< T, R, C >
- run()
  : Eigen::internal::general\_matrix\_matrix\_product< Index, stan::agrad::var, LhsStorageOrder, ConjugateLhs, stan::agrad::var, RhsStorageOrder, ConjugateRhs, ColMajor >
  , Eigen::internal::general\_matrix\_vector\_product< Index, stan::agrad::var, ColMajor, ConjugateLhs, stan::agrad::var, ConjugateRhs >
  , Eigen::internal::general\_matrix\_vector\_product< Index, stan::agrad::var, RowMajor, ConjugateLhs, stan::agrad::var, ConjugateRhs >
  , Eigen::internal::significant\_decimals\_default\_impl< stan::agrad::var, false >


---

[ Stan Home Page ]

*© 2011–2014,
Stan Development Team.*
